# Supplementary material for: Descriptive study of chest x-ray examination in mandatory annual health examinations at the workplace in Japan
Source: PLoS One. 2022 Jan 12;17(1):e0262404. doi: 10.1371/journal.pone.0262404 (PMC8754336; doi:10.1371/journal.pone.0262404)
Supplement: S1 Table — (DOCX) [file pone.0262404.s001.docx]

| S1 Table. Expected morbidity of tuberculosis and lung cancer based on age–sex distribution of 42 institutions associated with NFHA* | | | | | | | | | | |
| --- | --- | --- | --- | --- | --- | --- | --- | --- | --- | --- |
|  | Institutions with NFIHO | | New tuberculosis patients per 100,000 persons^1^ | | New lung cancer patients per 100,000 persons^2^ | | Estimated incidence of tuberculosis | | Estimated incidence of lung cancer | |
| Age | Men | Women | Men | Women | Men | Women | Men | Women | Men | Women |
| 20–24 | 243,340 | 193,942 | 12.1 | 8.2 | 0.2 | 0.3 | 29.6 | 16.0 | 0.5 | 0.5 |
| 25–29 | 292,975 | 169,298 | 9.9 | 9.1 | 0.8 | 0.8 | 28.9 | 15.3 | 2.3 | 1.4 |
| 30–34 | 325,618 | 157,115 | 7.3 | 6.9 | 1.3 | 1.3 | 23.9 | 10.8 | 4.2 | 2.0 |
| 35–39 | 332,681 | 173,491 | 6.7 | 5.3 | 4.2 | 3.9 | 22.1 | 9.3 | 14.0 | 6.7 |
| 40–44 | 378,963 | 225,382 | 6.7 | 5.0 | 8.8 | 6.9 | 25.6 | 11.3 | 33.3 | 15.5 |
| 45–49 | 347,184 | 221,463 | 8.7 | 5.4 | 18.3 | 12.9 | 30.1 | 12.0 | 63.5 | 28.5 |
| 50–54 | 284,574 | 188,693 | 10.3 | 4.9 | 40.9 | 24.1 | 29.3 | 9.3 | 116.4 | 45.6 |
| 55–59 | 252,547 | 165,223 | 12.7 | 5.7 | 85.7 | 43.7 | 32.0 | 9.4 | 216.4 | 72.2 |
| 60–64 | 211,314 | 122,245 | 15.4 | 6.1 | 167.2 | 71.3 | 32.6 | 7.4 | 353.3 | 87.2 |
| 65–69 | 125,331 | 67,357 | 19.3 | 7.2 | 278.5 | 111.3 | 24.2 | 4.8 | 349.0 | 75.0 |
| 70–74 | 38,757 | 21,269 | 27.8 | 11.3 | 412.3 | 148.1 | 10.8 | 2.4 | 159.8 | 31.5 |
| ≥75 | 29,304 | 38,881 | 44.1 | 19.9 | 490.5 | 171.5 | 12.9 | 7.7 | 143.7 | 66.7 |
| Total | 2862588 | 1744359 |  |  |  |  | 301.9 | 115.8 | 1456.6 | 432.6 |
| Expected morbidity per 100,000 persons | | | | |  |  | 10.5 | 0.7 | 50.9 | 24.8 |
| Expected morbidity per 100,000 persons (95% confidence interval) | | | | |  | Total | 9.1 (8.2–9.9) | | 41.0 (39.2–42.9) | |
| ^1^The Tuberculosis Research Institute. The Tuberculosis Surveillance Center. http://www.jata.or.jp/rit/ekigaku/. Accessed April 8, 2020. [Reference 4] | | | | | | | | | | |
| ^2^Cancer Registry and Statistics. Cancer Information Service, National Cancer Center, Japan. Monitoring of Cancer Incidence in Japan (MCIJ). [Reference 5] | | | | | | | | | | |
| *National Federation of Industrial Health Organization | | | | |  |  |  |  |  |  |
