# Supplementary material for: Descriptive study of chest x-ray examination in mandatory annual health examinations at the workplace in Japan
Source: PLoS One. 2022 Jan 12;17(1):e0262404. doi: 10.1371/journal.pone.0262404 (PMC8754336; doi:10.1371/journal.pone.0262404)
Supplement: S2 Table — (DOCX) [file pone.0262404.s002.docx]

| S2 Table. Results of recent low-dose CT examinations in 2016–2018 | | | | | | | |  |  |  |  |  |  |  |  |  |
| --- | --- | --- | --- | --- | --- | --- | --- | --- | --- | --- | --- | --- | --- | --- | --- | --- |
|  | Men | | | | Women | | | | Total | | | | New patients with lung cancer per 100,000 persons^1^ | | Estimated number of individuals with lung cancer | |
| Age | Examinees | Number of workers who required follow-up examinations | Number of workers who underwent follow-up examination | Diagnosis of lung cancer | Examinees | Number of workers who required follow-up examinations | Number of workers who underwent follow-up examination | Diagnosis of lung cancer | Examinees | Number of workers who required follow-up examinations | Number of workers who underwent follow-up examination | Diagnosis of lung cancer | Men | Women | Men | Women |
| 50–54 | 2277 | 17 | 15 | 1 | 236 | 5 | 5 | 0 | 2513 | 22 | 20 | 1 | 40.9 | 6.3 | 0.9 | 0.0 |
| 55–59 | 2382 | 15 | 15 | 2 | 296 | 1 | 1 | 0 | 2678 | 16 | 16 | 2 | 85.7 | 9.8 | 2.0 | 0.0 |
| 60–64 | 2296 | 33 | 26 | 0 | 314 | 4 | 3 | 0 | 2610 | 37 | 29 | 0 | 167.2 | 13.1 | 3.8 | 0.0 |
| 65–69 | 2001 | 25 | 22 | 0 | 376 | 2 | 2 | 0 | 2377 | 27 | 24 | 0 | 278.5 | 19.7 | 5.6 | 0.1 |
| 70–74 | 1174 | 19 | 14 | 0 | 138 | 3 | 3 | 0 | 1312 | 22 | 17 | 0 | 412.3 | 30.9 | 4.8 | 0.0 |
| ≥75 | 127 | 3 | 2 | 0 | 15 | 0 | 0 | 0 | 142 | 3 | 2 | 0 | 490.5 | 45.9 | 0.6 | 0.0 |
| Total | 10257 | 112 | 94 | 3 | 1375 | 15 | 14 | 0 | 11632 | 127 | 108 | 3 |  |  | 17.8 | 0.2 |
| Total | |  |  |  |  |  |  |  |  |  |  |  |  |  | 18.1 | |
| Expected morbidity per 100,000 persons (95% confidence interval) | | | |  |  |  |  |  |  |  |  |  |  |  | 155.2 (80.3–230.9) | |
| ^1^Cancer Registry and Statistics. Cancer Information Service, National Cancer Center, Japan. Monitoring of Cancer Incidence in Japan (MCIJ). [Reference 5] | | | | | | | | | | | | | | |  | |
