# Supplementary material for: Descriptive study of chest x-ray examination in mandatory annual health examinations at the workplace in Japan
Source: PLoS One. 2022 Jan 12;17(1):e0262404. doi: 10.1371/journal.pone.0262404 (PMC8754336; doi:10.1371/journal.pone.0262404)
Supplement: S3 Table — (DOCX) [file pone.0262404.s003.docx]

| S3 Table. Characteristics of workers who died of lung cancer | | | | |  |  |
| --- | --- | --- | --- | --- | --- | --- |
|  |  |  | History of LDCT screening |  |  |  |
| Case no. | Age, years | Sex |  | Smoking status | Pathology | Clinical stage |
| 1 | 50 | Male | Done | Past | Small cell | Unknown |
| 2 | 60 | Male | Done | Current | Small cell | Unknown |
| 3 | 50 | Male | Never | Current | Small cell | Unknown |
| 4 | 40 | Male | Never | Current | Unknown | Unknown |
| 5 | 50 | Male | Done | Past | Small cell | IV |
| 6 | 50 | Male | Done | Past | Small cell | Unknown |
| 7 | 60 | Male | Done | Current | Squamous | IV |
| 8 | 50 | Male | Never | Past | Unknown | Unknown |
| 9 | 50 | Male | Done | Past | Adenocarcinoma | Unknown |
| 10 | 40 | Male | Never | Current | Small cell | IV |
| 11 | 50 | Male | Never | Never | Adenocarcinoma | III |
| 12 | 40 | Female | Never | Never | Adenocarcinoma | Unknown |
| 13 | 30 | Male | Never | Past | Adenocarcinoma | IV |
| 14 | 60 | Male | Never | Current | Small cell | IV |
| 15 | 40 | Male | Never | Never | Adenocarcinoma | IIIB |
| 16 | 50 | Male | Never | Past | Unknown | IV |
| 17 | 50 | Male | Never | Past | Small cell | Unknown |
