# Supplementary material for: Descriptive study of chest x-ray examination in mandatory annual health examinations at the workplace in Japan
Source: PLoS One. 2022 Jan 12;17(1):e0262404. doi: 10.1371/journal.pone.0262404 (PMC8754336; doi:10.1371/journal.pone.0262404)
Supplement: S4 Table — (DOCX) [file pone.0262404.s004.docx]

| S4 Table. Diseases other than tuberculosis and lung cancer detected by chest x-ray examination | | | | | |
| --- | --- | --- | --- | --- | --- |
|  | Men | |  | Women | |
|  | Diagnosis | Number |  | Diagnosis | Number |
| 1 | Emphysema | 12 |  | Nontuberculous mycobacteriosis | 22 |
| 2 | Nontuberculous mycobacteriosis | 12 |  | Pneumonia | 13 |
| 3 | Pneumonia | 11 |  | Mediastinal tumor | 11 |
| 4 | Pneumothorax | 11 |  | Lung cyst | 9 |
| 5 | Bronchitis | 10 |  | Pneumothorax | 9 |
| 6 | Lung cyst | 10 |  | Inflammatory lung disease | 6 |
| 7 | Mediastinal tumor | 8 |  | Emphysema | 5 |
| 8 | Chronic bronchitis | 7 |  | Pulmonary fibrosis | 5 |
| 9 | Inflammatory findings | 6 |  | Sarcoidosis | 5 |
| 10 | Sarcoidosis | 6 |  | Benign tumor | 5 |
| 11 | Benign tumor | 6 |  | Bronchitis | 4 |
| 12 | Pulmonary fibrosis | 5 |  | Chronic bronchitis | 4 |
| 13 | Ground-glass opacity | 5 |  | Interstitial pneumonia | 4 |
| 14 | Interstitial pneumonia | 5 |  | Pneumoconiosis | 4 |
| 15 | Old pneumonia | 5 |  | Aortic aneurysm | 4 |
| 16 | Old pulmonary tuberculosis | 5 |  | Pleural thickening | 3 |
| 17 | Pneumoconiosis | 5 |  | Bronchiectasis | 3 |
| 18 | Right thyroid calcified nodules | 5 |  | Middle lobe syndrome | 2 |
| 19 | Aortic aneurysm | 4 |  | Chronic obstructive pulmonary disease | 2 |
| 20 | Organized pneumonia | 4 |  | Inflammatory scar | 2 |
