# Supplementary material for: Descriptive study of chest x-ray examination in mandatory annual health examinations at the workplace in Japan
Source: PLoS One. 2022 Jan 12;17(1):e0262404. doi: 10.1371/journal.pone.0262404 (PMC8754336; doi:10.1371/journal.pone.0262404)
Supplement: Attachment — Submitted filename: response_210615.docx [file pone.0262404.s005.docx]

**Reviewer #1:**

Dr. Watanabe analyzed the national occupational health examination database to appreciate the effectiveness of routine annual chest x-ray in Japan. The main findings were (1) Among the 4764985 participants who underwent chest x-ray examination for TB detection and follow-up data for diagnosis were available, 47962 subjects were advised for follow up, 16734 subjects underwent follow-up exam, and 88 patients were diagnosed of active TB (rate of detection 1.8/100000, positive predictive value 0.53%); (2) Among 3688395 participants who underwent chest x-ray for lung cancer detection, 38146 subjects were advised for follow-up examination, 14137 subjects underwent follow-up exam, and 334 cases were diagnosed with lung cancer (rate of detection 9.1/100000, positive predictive value 2.36%). (3) Stage I lung cancer accounted for 43.9% among the lung cancer revealed by annual chest x-ray examination. The authors concluded that chest x-ray examination plays a role in the detection of active TB and early stage lung cancer.

The study is well design and the manuscript is well written. However, I do not totally agree with the interpretation of the data presented. I would suggest some minor revisions.

**Response:** The authors would like to thank the reviewer for the feedback. We have made every effort to address the issues raised and to respond to all comments. The revisions are indicated in red color text in the revised manuscript. In addition, we have carried our English check by the native speakers again. Please, find next a detailed, point-by-point response to the comments below.

**Review１**

1. I am confused with the calculation of positive predictive value for TB detection and lung cancer. For the positive predictive value of chest x-ray to detect active TB, the authors use the number of cases diagnosed with tuberculosis (88) as the numerator and the number of participants who underwent follow-up (16734) as the denominator. Were all participants advised for follow-up examination due to suspicion of active TB? If yes, then the positive predictive value should be 88/47962 = 1.8%. If no, then the denominator should be the number of subjects whose chest x-ray were considered possible active TB. Similar issues existed in the calculation of positive predictive value of lung cancer detection.

**Response:** As pointed out by the reviewer, active TB was detected in 88 patients. Thus, the PPV was actually 0.18% (88/47962). However, we could follow up only 16734 (34.9%) participants who underwent follow-up examination. The measured PPV of 0.18% was the lowest PPV and might have been underestimated. The maximum PPV was calculated as 0.56% (88/16734), which might have been overestimated. Thus, plausible PPV was estimated to range between 0.18% and 0.56%.

We have provided this information in the revised manuscript as follows:

‘The positive predictive value (PPV) was primarily calculated to be 0.18% (88/47,962). This value might have been an underestimation, because the cases that could be followed up were 16,734 (34.9%). Thus, the maximum PPV was calculated as 0.56% (88/16734), which might have been an overestimation. Plausible PPV was estimated to range between 0.18% and 0.56%.’ (Lines 171–175)

Moreover, we have revised the data presented in Tables 2 and 3.

2. According to the estimation by the authors (line 172 of the manuscript), the expected incidence of active TB for the population studied was 9.1/100000. However, the rate of detection was 1.8/100000. That means more than 80% of the cases of active were not detected by annual chest x-ray examination. With an extremely low positive predictive value (0.53%), it is difficult to appreciate the usefulness of routine annual chest x-ray examination for the purpose of TB detection in countries with low prevalence of TB. The variability and reproducibility were well-known issues for chest x-ray interpretation. If an outbreak of clustered transmission among work place occurs, retrospective re-evaluation of the chest x-ray with a documented diagnosis in mind may reveal some obscured abnormalities and cause trouble to the radiologist who firsthand interpreted the chest x-ray. Similar issues existed in the interpretation of usefulness of lung cancer detection with chest x-ray. At least, these concerns should be listed.

**Response:**

We would like to thank the reviewer for the comment. Indeed, the estimated prevalence of tuberculosis was 9.1/100,000 persons, and the detection rate on chest X-rays was 1.8/100,000 persons. The estimated prevalence of lung cancer was 41.0/100,000 persons, while the detection rate was 9.1/100,000 persons. Since the detection rate of chest X-rays is considered to be limited, we have revised the corresponding part in the Discussion section as follows:

(Before editing)

Line 247-250: Considering the statistics from the national database and the natural course of TB, CXR examination in periodic workplace health examinations could be expected to actually play a role to some extent in the detection of active TB in clinical practice. However, the contribution of CXR to tuberculosis infection control might not be deemed essential.

Line 329-330: In conclusion, based on the actual rate of detection of TB using CXR, we ascertained that CXR plays a certain role in TB detection at the workplace.

(After editing)

‘Considering the national database statistics and the natural course of TB, it is difficult to appreciate the usefulness of routine annual CXR examination to detect TB in countries with low TB prevalence. Thus, the contribution of CXR to tuberculosis infection control might not be significant.’. (Lines 262–265)

‘In conclusion, based on the actual detection rate of TB using CXR, we ascertained that CXR plays a little role in TB detection in the general workplace. In addition, variability and reproducibility are well-known issues for CXR interpretation. If an outbreak of clustered transmission occurs in the workplace or if some LC cases are overlooked, retrospective re-evaluation of cases with a documented diagnosis who were previously evaluated using CXR may reveal discrepancies and create issues for the radiologist who interpreted the CXR’. (Lines 348–353)

3. The expected incidence of lung cancer increases exponentially in the elderly. I suggest the authors to extend the table 3 by further stratifying the detection of lung cancer among age groups of <40, 40-60, and >60 years old.

**Response:**

Please note that those who underwent CXR examination were aged ≤60 years, as the retirement age was at 60 years in 2016. Thus, the number of those aged >60 years was negligible.

4. The table 5 is not quite relevant to the topics of the present study. It is difficult to determine the accuracy, sensitivity, and specificity by chest x-ray examination for the diagnoses listed. I would suggest to remove the information of table 5 from the present report.

**Response:**

We would like to thank the reviewer for the constructive comment. According to the reviewer’s suggestions, we have revised our manuscript and removed the information presented in Table 5.

Examination of chest X-rays at workplace aimed to screen tuberculosis and lung cancer. Thus, this study included descriptive information regarding the observed stage of disease according to CXR findings. Therefore, we have moved this Table to the Supplementary files.

5. In line 256-258, the sentence “From the perspective of cost-effectiveness, screening for Tb should be considered when the prevalence is lower than 10 per 100,000 persons” is ambiguous.

**Response:**

We would like to thank the reviewer for the comment. Please note that we have revised this part as follows: ‘The World Health Organisation aims to reduce the new TB cases by approximately 90% by 2035 and reduce the prevalence to <10 per 100,000 population [19]. From the perspective of cost-effectiveness, active case-findings for TB using CXR should be considered among high-risk populations. Nevertheless, the strategy should be re-considered when the prevalence is <10 per 100,000 persons in Japan [20]. Active case-findings among all general workers may be unnecessary.’. (Lines 271–277)

**Reviewer #2:**

Numbers of diagnoses and stage are poor surrogate outcomes for the effect of health checks. Increased numbers of diagnosis and more localised stages can be and impact of a beneficial effect, a harmful effect (especially overdiagnosis) or both. Moreover, mortality rates in a non-randomised trial cannot be used as a result of benefits. Finally, survival rates will be biased due to lead time bias and leangt time bias (overdiagnosis bias) and are the not useful outcomes when it comes to early disease detection, e.g. screening and health checks.

**Response:**

The authors would like to thank the reviewer for the constructive feedback and suggestions to improve the manuscript. We have made every effort to address the issues raised and to respond to the reviewer’s comments. The revisions are indicated in red color text in the revised manuscript. In addition, we have carried our English check by the native speakers again.

We fully understood that the evaluation of screening such as cancer screening should be based on the first priority demonstrating its effectiveness as screening by randomized controlled trials. Many studies have already conducted studies on the effectiveness of chest X-rays on lung cancer screening. However, it is only an evaluation of the effectiveness of a screening method by experimental research protocols with limited sample size. Thus, to evaluate usefulness as a measure, a realistic evaluation when implemented in society is also necessary. The purpose of this study is to provide actual descriptive data on the current state of social implementation of the screening as a measure, not to evaluate the effectiveness of the screening. It only shows social significance.

Please note that we have added the following part to the Introduction section of the revised manuscript to address the issues raised by the reviewer and to clearly describe our purpose:

‘The effectiveness for screening diseases such as cancer should be determined with randomised controlled trials using mortality as the outcome. The Japanese guidelines for lung cancer screening recommend the performance of CXR [9]. However, these guidelines were developed based on the findings of experimental studies with a limited sample size that evaluated the effectiveness of screening methods. Thus, to evaluate significance as a measure, a realistic evaluation when implemented in a large population is also needed. The purpose of this study was to provide actual descriptive data on the current state of social implementation of screening, and not to evaluate the effectiveness of the screening method.’. (Lines 67–74)
