# Supplementary material for: Descriptive study of chest x-ray examination in mandatory annual health examinations at the workplace in Japan
Source: PLoS One. 2022 Jan 12;17(1):e0262404. doi: 10.1371/journal.pone.0262404 (PMC8754336; doi:10.1371/journal.pone.0262404)
Supplement: Attachment — Submitted filename: response_2021.11.25.docx [file pone.0262404.s006.docx]

**Reviewer #2:**

Numbers of diagnoses and stage are poor surrogate outcomes for the effect of health checks. Increased numbers of diagnosis and more localised stages can be and impact of a beneficial effect, a harmful effect (especially overdiagnosis) or both. Moreover, mortality rates in a non-randomised trial cannot be used as a result of benefits. Finally, survival rates will be biased due to lead time bias and leangt time bias (overdiagnosis bias) and are the not useful outcomes when it comes to early disease detection, e.g. screening and health checks.

**Response:**

The authors would like to thank the reviewer for the constructive feedback and suggestions to improve the manuscript. We have made every effort to address the issues raised and to respond to the reviewer’s comments. The revisions are indicated in red font in the revised manuscript.

Please note that we have revised the following part to the Abstract, Introduction, and Discussion section of the revised manuscript to address the issues raised by the reviewer and to describe our purpose clearly:

As the reviewer mentioned, the numbers of the detection and early-stage cancer are generally not useful outcomes, mainly because they can include cases with overdiagnosis. Particularly the problem of LDCT-based lung cancer screening is overdiagnosis. Thus, the effectiveness for screening diseases such as cancer must be determined with randomised controlled trials using mortality as the outcome. Lung cancer screening with CXR is conducted based on evidence of the effect on reduction of mortality. However, in CXR, the problem of sensitivity is greater than the problem of overdiagnosis in terms of screening efficacy. The purpose of this study was to provide actual descriptive data on the current state of social implementation of screening among workers and not to evaluate the effectiveness of the screening method.

Our study investigated the detection rate and early cancer detection rate in medical examinations that have already been implemented as a law in the workplace for public health.

Therefore, we revised our interpretation and conclusion according to reviewer’s comments.

This revision appears in red font.

**Abstract**

Before:

L17-18:

This study aimed to assess the clinical utility of chest X-ray examination in the workplace and provide basic data to consider future policies for mandatory annual health examinations.

Revised:

L17-18:

This study aimed to provide basic data to consider future policies for mandatory annual health examinations in the workplace.

Before;

L36-38

Early-stage lung cancer can also be detected among workers by Chest x-ray examinations. However, low-dose computed tomography may be superior for lung cancer screening because it can detect twice as many cases as chest radiography.

Revised:

L34-38:

With regard to LC screening, the detection rate of LC was lower, approximately 50%, than the expected rate (41.0 per 100,000 persons) of LC morbidity based on the age-sex distribution of this study population. However, the role of CXR for LC screening cannot be mentioned based on this result because assessment of mortality reduction is essential to evaluate.

**Introduction**

Before:

L66-73

The effectiveness for screening diseases such as cancer should be determined with randomised controlled trials using mortality as the outcome. The Japanese guidelines for lung cancer screening recommend the performance of CXR [9]. However, these guidelines were developed based on the findings of experimental studies with a limited sample size that evaluated the effectiveness of screening methods. Thus, to evaluate significance as a measure, a realistic evaluation when implemented in a large population is also needed. The purpose of this study was to provide actual descriptive data on the current state of social implementation of screening, and not to evaluate the effectiveness of the screening method.

Revised:

L66-74

Japanese guideline for lung cancer screening recommends CXR [9]. Thus mandatory CXR in workplace plays a role in lung cancer screening in addition to screening for TB. The first priority of evaluation of effectiveness on cancer screening must be based on randomized controlled trials (RCT) using mortality as an outcome. Theoretically, the detection number of cancers is not a useful outcome to evaluate effectiveness because cancers detected by screening can include cases with overdiagnosis. Thus this study did not aim to evaluate the effectiveness of CXR for cancer screening. The purpose of this study is to provide actual descriptive data on the current state of social implementation of the screening as a measure, not directly to assess the effectiveness of CXR for cancer screening.

**Discussion**

Before:

L293-294

Merely by considering this result, the early-stage detection rate with LDCT was two times higher than that by CXR.

Revised:

L287-293

However, outcomes for the effect of cancer screening must be addressed by mortality, but not by increased numbers of diagnoses and more localized stages, because cancer screening has several biases and harmful effects such as overdiagnosis [23,24]. Regarding LC screening, the main problem of CXR examination was reported to be a low sensitivity while that of LDCT was overdiagnosis [25]. Thus, using our data on this study, we cannot compare or evaluate the screening effects between CXR and LDCT. We only provided descriptive data as reference values.

**References**

Three references have been added.

23.	Brawley OW, Kramer BS. Cancer screening in theory and in practice. J Clin Oncol 2005;23:293-300.

24.	Raffle A, Gray M. Screening: evidence and practice. Oxford University Press, 2007.

25.	T Sobue 1, T Suzuki, M Matsuda, T Horai, A Kajita, K Kuriyama, M Fukuoka, Y Kusunoki, M Kikui, S Ryu, et al. Sensitivity and specificity of lung cancer screening in Osaka, Japan. Jpn J Cancer Res. 1991;82(10):1069-76.

Before:

L298-299

‘’Moreover, this finding indicated that the utility of LDCT for screening for LC was almost twice as high as that of CXR.’’

Revised:

This sentence has been deleted.

Before:

L337-338

We could not effectively compare the usefulness of CXR and LDCT.

Revised:

L335-339

This is because all CXR in Japan has historically been started and performed as TB screening for workers. The number of detections as cancer screening cannot be evaluated due to problems of harmful effects such as overdiagnosis. Thus, we could not effectively compare the usefulness of CXR and LDCT only based on our results.

Before:

L351-358

With regard to LC screening, the detection rate with CXR was lower than that with LDCT, and the detection rate in Stage I using CXR, when detection may save lives, was approximately 50% lower than that using LDCT. These findings were consistent with those of a previous residence-based comparative study by Nawa et al [23]. Nevertheless, our results do not support the recommendation for the exclusion of CXR from the mandatory annual health examinations; however, effective introduction of LDCT screening to workplace health examinations should be considered as a substitute for CXR for LC screening in the future.

Revised:

L352-359

With regard to LC screening, the detection rate with CXR was lower, approximately 50%, than the expected rate of LC morbidity based on the age-sex distribution of this study population. However, the results of this study do not evaluate the actual utility of CXR in the mandatory annual health examinations. Further follow-up studies are needed to assess mortality reduction in workplace for LC screening. Inductions of LDCT are discussed for LC screening worldwide, the effective introduction of LDCT screening to workplace health examinations should be considered in the future, based on the evidence by RCT in Japan.
