# Supplementary material for: Descriptive study of chest x-ray examination in mandatory annual health examinations at the workplace in Japan
Source: PLoS One. 2022 Jan 12;17(1):e0262404. doi: 10.1371/journal.pone.0262404 (PMC8754336; doi:10.1371/journal.pone.0262404)
Supplement: Attachment — Submitted filename: response_2021.12.19.docx [file pone.0262404.s007.docx]

**Reviewer #1:**

In the Conclusion section of this revised version. The sentence is ambiguous: "Inductions of LDCT are discussing for LC screening worldwidely...". Please revise it for clarity.

**Response:**

We would like to appreciate the Reviewers and the Editorial Board for taking the time to review our manuscript and thank their constructive and thoughtful comments. According to the suggestion, we have revised our manuscript below.

**Conclusion**

Before:

L357-359:

Inductions of LDCT are discussing for LC screening worldwidely, effective introduction of LDCT screening to workplace health examinations should be considered in the future, based on the evidence by RCT in Japan.

Revised:

L357-362:

Reports from North America have shown a significant reduction of mortality regarding LDCT screening, and it will be considered in high-risk populations, such as those over 50 years with heavy smokers in workplaces. Our study, however, demonstrated that the effectiveness of LDCT screening was limited for patients with small cell carcinoma. Thus, strengthening smoking cessation measures in workplaces should be considered as well as screening.
